# Improved Diagnosis of the Transition to *JAK2* ^V617F^ Homozygosity: The Key Feature for Predicting the Evolution of Myeloproliferative Neoplasms

**DOI:** 10.1371/journal.pone.0086401

**Published:** 2014-01-27

**Authors:** Mariana Selena Gonzalez, Carlos Daniel De Brasi, Michele Bianchini, Patricia Gargallo, Carmen Stanganelli, Ilana Zalcberg, Irene Beatriz Larripa

**Affiliations:** 1 Instituto de Medicina Experimental (IMEX), National Research Council (CONICET)–Academia Nacional de Medicina (ANM), Buenos Aires, Argentina; 2 Instituto de Investigaciones Hematológicas (IIHEMA), ANM, Buenos Aires, Argentina; 3 Centro de Investigaciones Oncológicas-Fundación Cáncer (CIO-FUCA), Instituto Alexander Fleming, Buenos Aires, Argentina; 4 Molecular Biology, Laboratory, Instituto Nacional do Câncer, Rio de Janeiro, Brazil; B.C. Cancer Agency, Canada

## Abstract

Most cases of BCR-ABL1-negative myeloproliferative neoplasms (MPNs), essential thrombocythemia, polycythemia vera and primary myelofibrosis are associated with *JAK2* ^V617F^ mutations. The outcomes of these cases are critically influenced by the transition from *JAK2* ^V617F^ heterozygosity to homozygosity. Therefore, a technique providing an unbiased assessment of the critical allele burden, 50% *JAK2* ^V617F^, is highly desirable. In this study, we present an approach to assess the *JAK2* ^V617F^ burden from genomic DNA (gDNA) and complementary DNA (cDNA) using one-plus-one template references for allele-specific quantitative-real-time-PCR (qPCR). Plasmidic gDNA and cDNA constructs encompassing one PCR template for *JAK2* ^V617F^ spaced from one template for *JAK2^Wild Type^* were constructed by multiple fusion PCR amplifications. Repeated assessments of the 50% JAK2^V617F^ burden within the dynamic range of serial dilutions of gDNA and cDNA constructs resulted in 52.53±4.2% and 51.46±4.21%, respectively. The mutation-positive cutoff was estimated to be 3.65% (mean +2 standard deviation) using 20 samples from a healthy population. This qPCR approach was compared with the qualitative ARMS-PCR technique and with two standard methods based on qPCR, and highly significant correlations were obtained in all cases. qPCR assays were performed on paired gDNA/cDNA samples from 20 MPN patients, and the *JAK2* ^V617F^ expression showed a significant correlation with the allele burden. Our data demonstrate that the qPCR method using one-plus-one template references provides an improved assessment of the clinically relevant transition of *JAK2* ^V617F^ from heterozygosity to homozygosity.

## Introduction

The discovery of a mutation in the Janus kinase 2 (*JAK2*) gene opened a new era in the understanding of *BCR-ABL-* negative myeloproliferative neoplasms (MPNs) [1]–[5]. An acquired transversion in *JAK2* exon 14 (c.1849G>T) that is confined to hematopoietic cells and results in p.Val617Phe (*JAK2* ^V617F^) is observed in approximately 90% of patients with polycythemia vera (PV), 50% of essential thrombocythemia (ET) cases and 50% of primary myelofibrosis (PMF) cases [1], [3]. *JAK2* ^V617F^ impacts the function of the pseudokinase JH2 domain, which normally plays a role in the auto-inhibition of *JAK2* kinase activity [4]. *In vitro* studies have demonstrated that *JAK2* ^V617F^ leads to a specific phosphorylation associated with the constitutive activation of the tyrosine kinase function [3].

Primarily involved in myeloid development, the JAK2 protein is a non-receptor tyrosine kinase associated with the cytoplasmic regions of several cytokine membrane receptors [6]. JAK2 is activated when these receptors bind to hematopoietic growth factors, and it acts as a molecular intermediary through the constitutive activation of *STAT5-*, *AKT-* and *ERK*-dependent pathways [7], [8].

After the acquisition of *JAK2* ^V617F^, loss of heterozygosity (LOH) may occur by the duplication of the mutant allele via mitotic recombination of the short arm of chromosome 9, resulting in homozygosity. Consequently, the quantity of mutant versus wild-type *JAK2* may vary significantly, introducing the concept of allele burden. The term homozygosity is employed to indicate patients in whom the level of mutant allele in the test sample is greater than 50% of the total *JAK2* (mutant [MT] plus wild type [WT]). The *JAK2* ^V617F^ burden has been correlated with changes in clinical phenotype and disease complications, such as thrombosis and myelofibrosis [9]–[11]. Homozygosity is associated with a significantly longer duration of disease, treatment with cytoreductive therapy and a higher rate of complications [3]. *JAK2* ^V617F^ LOH has been observed in approximately 30% of patients with PV and PMF, compared to only 2–4% of patients with ET [12], [13].

Therefore, the accurate estimation of the V617F allele burden and (in particular) the unbiased assessment of the 50% allele burden has gained major clinical relevance in patients with PV, ET and PMF because values significantly greater than 50% guarantee the presence of at least some cells exhibiting LOH and the prognostic consequences associated with this condition. The current methods to analyze the *JAK2* ^V617F^ allele burden are based on the absolute or disconnected quantification of standards for the MT and WT alleles. Hence, a practical approach to measure the V617F allele burden with a special focus on the accurate assessment of the one-plus-one MT:WT allelic ratio and the associated experimental error is highly desirable in this field.

This work presents a new approach to assess the *JAK2* ^V617F^ allele burden in gDNA (genomic DNA) and cDNA (complementary DNA from total RNA) samples using one-plus-one template references in a general strategy of allele-specific quantitative real time-PCR (qPCR).

## Materials and Methods

### Studied Population and Samples

Peripheral blood samples were obtained from a total of 53 patients with MPNs and 20 healthy donors (control group). Twenty of the MPN patients were diagnosed according to the current hematological criteria established by the World Health Organization (WHO) as six PV, five ET and nine PMF cases; these patients were used to test the allele burden and transcript expression of *JAK2* ^V617F^ for correlation analysis. Another group of 33 cases was used to validate the above method by comparing it with ARMS-PCR, and with two other standard qPCR assays. This study was approved by the local Institutional Ethics Committee (*Academia Nacional de Medicina de Buenos Aires*). Written informed consent was obtained in all cases. The patients’ characteristics are listed in Table 1.

**Table 1:** Patient characteristics.

	PV (n = 6)	ET (n = 5)	MF (n = 9)
Males/females	3/3	2/3	3/6
Median age (years)	64	58	55
Range age (years)	42–90	50–90	50–68
Characteristics at diagnosis:			
Hematocrit values (%)	57.2±2.3	42.2±2.3	33±0.9
White blood cells, ×10^9^/L	11.5±2	8.9±1.2	10.5±2
Neutrophils (%)	65.8±6,2	59±5	62.3±7.2
Platelets, x 10^9^/L	354.2±73.9	2943±2100	234.1±50.4
Splenomegaly	1/6	0/6	4/9
Patients on cytoreductivetreatment	4/6	3/5	6/9

gDNA and total RNA were extracted from total leukocytes by standard procedures after 3 cycles of lysing red cells from peripheral blood samples. Leukocyte pellets were either treated with TRIzol (Invitrogen, Argentina) for total RNA extraction or with phenol/Tris-HCl (pH: 8) for gDNA extraction. One microgram of total RNA was reverse transcribed into cDNA using random hexamer primers and reverse transcriptase M-MLV (Promega, Biodynamics Argentina). In addition, a gDNA sample from SET-2, a cell line derived from a MPN patient with *JAK2^V617F^* heterocigosity, was used to confirm the inaccurateness of using *JAK2^V617F^* positive cell lines as standards.

### Construction of *JAK2* ^V617F^-*JAK2* ^Wild Type^ (*JAK2* ^MT^-*JAK2* ^WT^) One-plus-one Template Reference Plasmids

**Figure 1:**
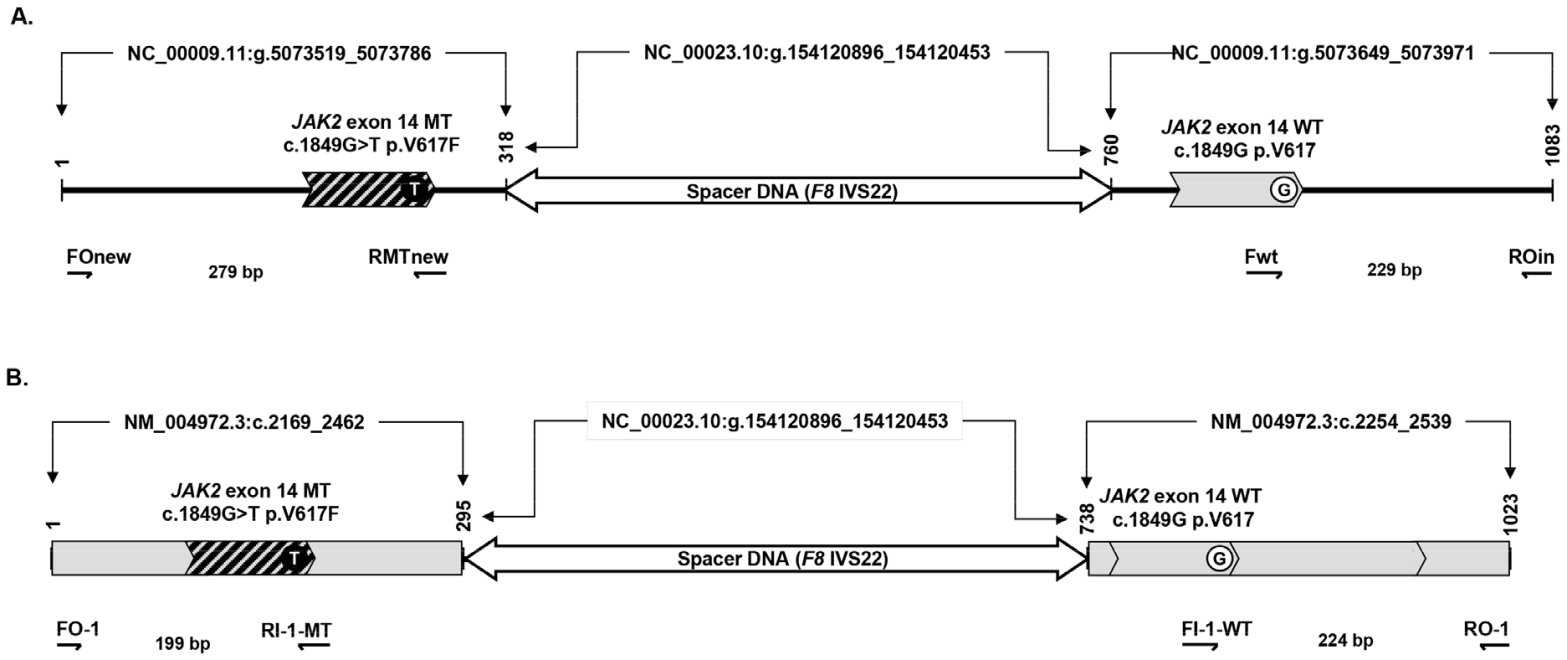
*JAK2* MT:WT 1∶1 reference constructs. Upper diagram: reference construct from genomic DNA. Bottom diagram: reference construct from complementary DNA. These diagrams show the allele-specific primers used to amplify the mutated allele (MT) and the wild-type allele (WT). Indicated are the GenBank files and the molecular sizes of each segment and of each amplification product.

The *JAK2* gDNA-MT::WT 1::1 and *JAK2* cDNA-MT::WT 1::1 reference constructs consisted of a tripartite structure (i.e., an MT-left arm, a spacer and a WT-right arm) (Figure 1A and 1B). Each construct provided two templates for qPCR amplification: one for *JAK2^V617F^* and one for *JAK2 ^WT^*. These constructs were assembled following a strategy of multiple fusion PCR amplifications with conventional primers and specially designed fusion oligonucleotides (Table 2), as described in detail in Methods S1 and Figure S1.

**Table 2:** Oligonucleotide primers.

Name	Sequence (5′->3′)	Reference
*Construction of cDNA MT:WT 1::1 template reference*		
FO-1	ATTTTTAAAGGCGTACGAAGAGAAGTAG	(14)
RO-1	ATAAGCAGAATATTTTTGGCACATACAT	(14)
Up-Sp-cDNA	GAAGTTGCTAAACAGaagaaaccccaggaaacaga	This study.
Lo-Sp-cDNA	CTGAATAGTTTCTGTctcagcccctaagtcgtatc	This study.
*Construction of gDNA MT:WT 1::1 template reference*		
FOnew	CATATAAAGGGACCAAAGCACA	This study.
FOin	TCCTCAGAACGTTGATGGCAG	(15)
ROin	ATTGCTTTCCTTTTTCACAAGAT	(15)
Up-Sp-gDNA	CAGAGCATCTGTTTTaagaaaccccaggaaacaga	This study.
Lo-Sp-gDNA	GACTGTTGTCCATAActcagcccctaagtcgtatc	This study.
*Allele specific cDNA primers*		
RI-1	ACCAGAATATTCTCGTCTCCACAaAA	(14)
FI-1	GCATTTGGTTTTAAATTATGGAGTATaTG	(14)
*Allele specific gDNA primers*		
Rmt	GTTTTACTTACTCTCGTCTCCACAaAA	(15)
Fwt	GCATTTGGTTTTAAATTATGGAGTATaTG	(15)
RMTnew	TTACTTACTCTCGTCTCCACAaAA	This study.

For the amplification and storage of the qPCR amplification references, the cDNA and gDNA MT-WT one-plus-one template PCR products were cloned into plasmid vector pCR2.1-TOPO (Invitrogen SRL, Argentina) (details of the procedure are provided in the last section of Methods S1).

The cDNA and gDNA *JAK2* ^V617F^ *-JAK2^WT^* one-plus-one template reference plasmids are available for research use only after a Material Transfer Agreement (MTA) form is signed.

### Confirmation of the Uniqueness of *JAK2* ^V617F^ in both the gDNA and cDNA Constructs by BsaXI Restriction Analysis and DNA Sequencing

The *JAK2* ^V617F^ mutation (c.1849G>T) introduces a single BsaXI restriction site in both gDNA and cDNA constructs. To investigate the presence of a single copy of mutated *JAK2* in each construct, BsaXI restriction analysis was performed. Three microliters of PCR products obtained from an aliquot of a 10^−3^ dilution of the gDNA plasmid with primers FOin and ROin, as well as 3 µL of PCR products from a 10^−7^ dilution of the cDNA plasmid with primers FO-1 and RO-1, were subjected to BsaXI restriction with 20 units of enzyme in a total volume of 20 µl under the conditions recommended by the manufacturer (New England Biolabs, USA). The restriction products were analyzed using EtBr-stained agarose gel electrophoresis (2%), Figure S2 (E).

In addition, the *JAK2* constructs (gDNA and cDNA MT::WT 1::1) were bidirectionally sequenced (with FOin and ROin for the gDNA construct and with FO-1 and RO-1 for the cDNA construct) using the fluorescently labeled chain termination approach (BigDye ABI, Argentina) and an ABI 3130 XL apparatus (Genetic Analyzer from Applied Biosystems). The DNA sequences of MT-arm and WT-arm from the gDNA and cDNA constructs are shown in Figure S2 C and D, respectively.

### Primer Specificity and Structures of *JAK2* gDNA and cDNA Reference Plasmids

The molecular structures of the gDNA and cDNA reference plasmids were studied using PCR amplification experiments with multiple primer pair combinations (Table 2). Two different annealing temperatures (58°C and 60°C) were evaluated, and 2 µl from a 10^−7^ dilution of the gDNA and cDNA plasmids was amplified. The following optimized PCR thermocycling protocol was applied: an initial step of 94°C for 2 min; 25 cycles of 94°C for 30 sec, 58°/60°C for 45 sec and 72°C for 1 min, and a final extension step at 72°C for 5 min.

The desired specific structures of the gDNA and cDNA constructs (Figure 1A and 1B) were positively confirmed by the results shown in Figure S3 A and B, respectively. The results demonstrated that only the properly oriented primers produced size-specific PCR amplifications: FOn/RMTn, UpSp-g/LoSp-g and Fwt/ROin for the gDNA plasmid; and FO-1/RI-1, UpSp-c/LoSp-c and FI-1/RO-1 for the cDNA plasmid.

### Quantitative Real-time PCR

Quantitative real-time PCR (qPCR) was performed using the LightCycler 2.0 (Roche Diagnostics, Mannheim, Germany), which is based on SYBR Green chemistry. The 20-µl qPCR reaction mixtures contained 5 µl of sample cDNA or 40 ng of gDNA, 1X PCR Mix (LC FastStart DNA Master SYBR Green I, Roche Diagnostics, Argentina), 3.5 mM MgCl_2_ and 0.25 µM of each primer.

The optimal reaction conditions for amplifying *JAK2* ^V617F^ and *JAK2^WT^* from cDNA templates were 50 cycles of a 4-step PCR (95°C for 5 sec, 58°C for 3 sec, 72°C for 20 sec and 75°C for 1 sec). The optimal conditions for gDNA templates were 45 cycles of a 4-step PCR (95°C for 5 sec, 62°C for 6 sec and 72°C for 12 sec) after an initial denaturation (95°C for 10 min). The allele-specific primer sets used in this study to perform the relative quantification of *JAK2* ^V617F^ and *JAK2^WT^* from the patient cDNA samples were previously published by Vannucchi *et al.* [14], and the allele-specific primer sets for quantification from patient gDNA samples were modified from a qualitative ARMS-PCR strategy published by Jones *et al.* [15] (Table 2).

Calibration curves were generated using serial dilutions of the cDNA and gDNA *JAK2* ^V617F^::JAK2^WT^ 1::1 reference plasmids to estimate the qPCR amplification efficiencies and to quantify the *JAK2* ^V617F^ and *JAK2^WT^* alleles on gDNA and transcripts within the dynamic range.

### Quantification Strategy, Formulas and Error Estimation

The allele burden (AB) magnitude was calculated with the formula MT/(MT+WT) and expressed in arbitrary units related to the dilutions of the MT::WT 1::1 template reference curves. The errors associated with the MT and WT measurements were estimated using a linear regression (data not shown) between the mean and the standard deviation (SD) of each reference template dilution triplicate: 10^−5^, 10^−6^, 10^−7^ and 10^−8^ (Figure S4). To calculate the AB error (ΔAB) (in SD), the estimated MT and WT SDs were propagated using Gauss’ method of partial derivatives, i.e., ΔAB^2^ = |δAB/δMT|^2^ΔMT^2^+|δAB/δWT|^2^ΔWT^2^, which resulted in ΔAB_ (MT; ΔMT; WT; ΔWT)_ = [(WT * ΔMT)^2^+(MT*ΔWT)^2^]^½^/(MT+WT)^2^.

### *JAK2* ^V617F^ Genotyping by the Amplification Refractory Mutation System (ARMS)

Genomic DNA was extracted from total peripheral blood leucocytes obtained from 20 patients with suspected diagnoses of MPNs using phenol-chloroform according to standard procedures. The *JAK2* ^V617F^ ARMS (amplification refractory mutation system) analysis was performed using a multiplex PCR strategy, as described by Jones *et al.* [15]. The allele-specific primers contained a mismatch three bases from the 3′ end to maximize allele discrimination. The ARMS-PCR assay was performed using Taq DNA polymerase (Promega, Argentina), 25 ng of genomic DNA substrate and 30 amplification cycles (including a critical annealing temperature of 60°C) under standard amplification conditions. The results were analyzed by agarose gel electrophoresis (3%).

### Independent *JAK2* ^V617F^ Quantification Methods for Validation of the One-plus-one Reference System

Two independent methods were applied to validate our one-plus-one plasmid-based reference system by use of the Pearson correlation statistics. First, a qPCR system based on allele specific Taqman-probe quantification was performed as described Bousquet *et al* [16]. A second qPCR system based on allele specific amplification, which take advantage of human gDNA samples from a MPN patient with *JAK2^V617F^* homozygosity and a healthy blood donor with *JAK2^WT^* genotype to achieve the standard curves for qPCR, was performed as it is applied in a number of laboratories worldwide [8]. In order provide accurate standard curves the amount of *JAK2* PCR template copy number in both gDNA samples (i.e., *JAK2^V617F^* and *JAK2^WT^*) was equaled by experiments of PCR amplification analysis on a common reference region in *ABL1* exon 3.

## Results

### Strategy to Assess the *JAK2* ^V617F^ Allele Burden Using One-plus-one Template References

The *JAK2* ^V617F^ allele burden (AB) percentage (i.e., AB% = 100×MT/[MT+WT]) represents a weighted average of cells with zero, one or two copies of *JAK2* ^V617F^ in a given gDNA sample (ABg). The ABg% is largely similar for cDNA samples (ABc) but may be modified by differential *JAK2* allele mRNA expression, which is either produced by differential transcription rates of MT and WT or differential mRNA stability. In addition, the eventual contribution of allelic mRNA from enucleated elements in the whole blood samples (e.g., platelets) may introduce another source of variation to the ABc measurements.

ABg and ABc have no units because the units of MT and WT are equal (arbitrary units associated with the copies of the 1∶1 reference plasmid) and, therefore, cancelled. This is not the case when using two independent reference plasmids (MT and WT), whose accuracy in assessing the relative ABg relies upon two independent absolute copy number estimations. Hence, the main objective behind applying a one-plus-one template reference strategy is to reduce the inevitable biases associated with assessing *JAK2* ^V617F^ AB to approximately 50%, considering that this value is of major clinical significance.

**Figure 2:**
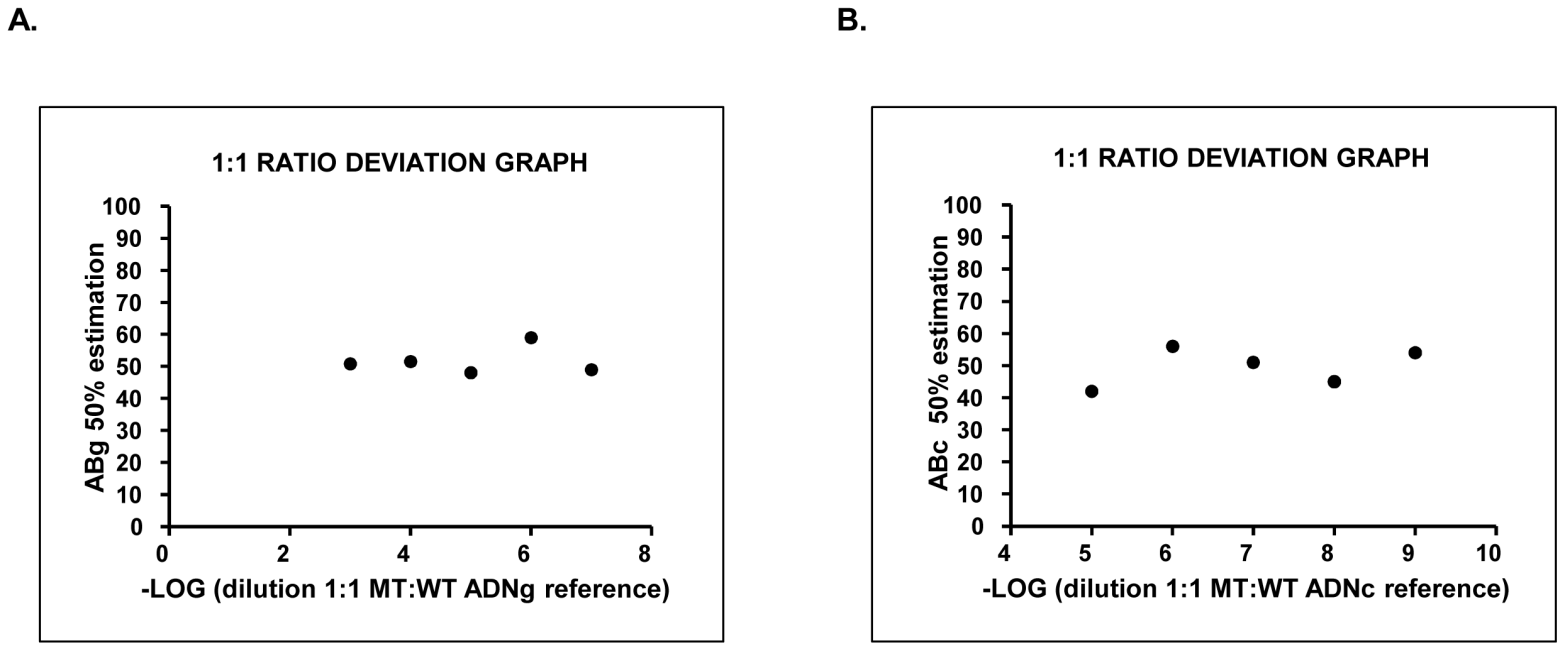
Graphics of deviation from the 1∶1 ratio in the dynamic range of dilutions of the reference plasmids. Left panel, gAB values for each plasmid dilution of gDNA; right panel, gAB values for plasmid cDNA.

The capacity of the gDNA and cDNA reference plasmids to assess ABg and ABc was investigated by repeated measurements of the same reference plasmid dilution within the dynamic range (defined as the range in which the fluctuation of 50% AB was minimal) (Figure 2). The ABg reference plasmid exhibited a mean of 52.53% and a standard deviation of 4.20% in the range 10^−3^–10^−7^ dilution (Figure 2A). Therefore, a limit value of *JAK2* ^V617F^ ABg of 56.73% (mean+SD) was predetermined to indicate a reliable transition to homozygosity. ABc exhibited a mean of 51.46% and a standard deviation of 4.21% in the range 10^−6^–10^−9^ (Figure 2B).

The dynamic range of the ABg analysis, reference plasmid dilutions with minimal errors (10^−3^–10^−7^) that corresponded to approximately 1.2×10^6^–1.2 copies, included the average *JAK2* copy number in gDNA inputs of 20 ng (6×10^3^ copies), which was used in our qPCR system. Although the dynamic range of ABc (10^−6^–10^−9^) corresponded to 9.65×10^3^–9.65 *JAK2* template copies, the difficulty in estimating the absolute *JAK2* template copies in the cDNA samples prevented a determination of whether the cDNA dynamic range actually contained the absolute template copy number.

Individual values of MT and WT were associated with an intrinsic operative error (SD), which was obtained by interpolating these MT and WT values by a linear regression performed with the reference plasmid dilution triplicates (plasmid dilutions versus SD triplicates). The propagation of these MT and WT errors in the allele burden formula (Materials and Methods) allowed the provision of each AB measurement with its corresponding experimental SD.

### Experimental Cutoff for Detecting *JAK2* ^V617F^ Positive Samples

In addition, to determine the experimental cutoff for discriminating *JAK2* ^V617F^-positive from -negative samples using qPCR, we assessed the ABg values from 20 healthy donors and obtained a mean value of 1.04% and an SD of 1.3%. A reliable *JAK2* ^V617F^ cutoff was based on an ABg threshold of 3.65%, which resulted from the mean plus two SD of the control population.

### Experimental Correlation between ARMS-PCR and qPCR Using One-plus-one Template References

**Figure 3:**
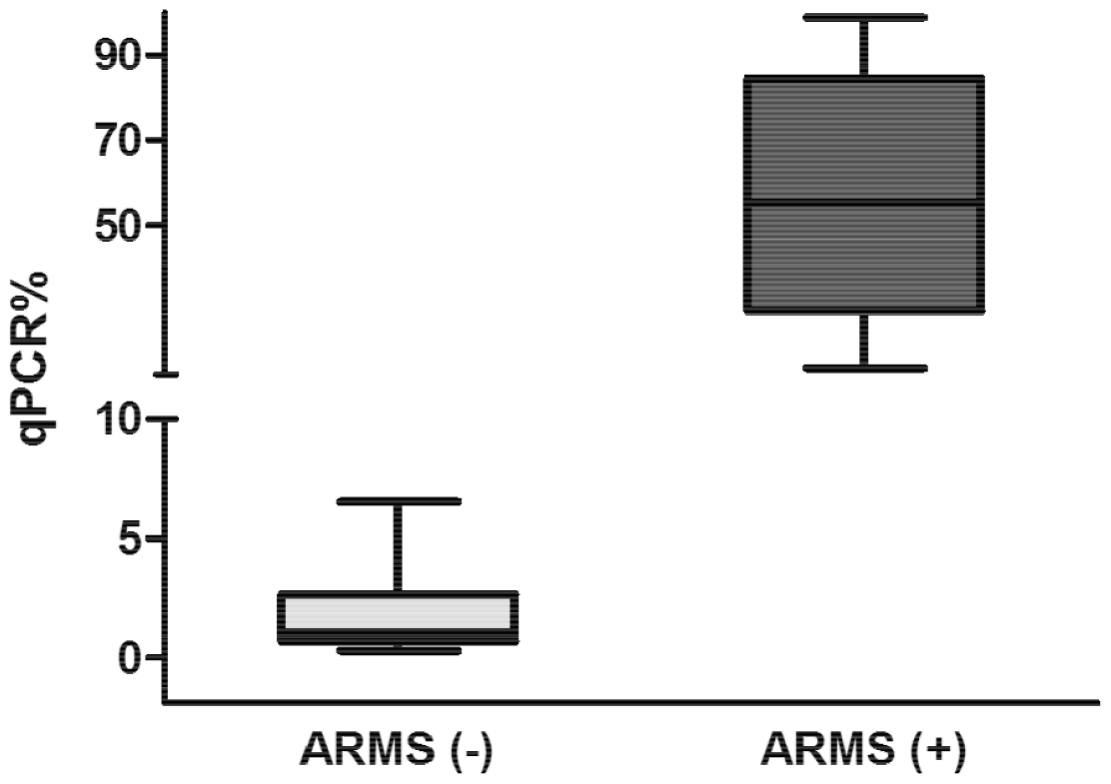
Comparison of ARMS-PCR and qPCR assays. Ten cases shown to be positive for the JAK2^V617F^ mutation using ARMS-PCR exhibited an allele burden of 55% ±9% (mean ± SE) according to qPCR. Ten negative cases according to ARMS-PCR showed an allele burden of 1.9% ±0.6%, including two cases that were negative by ARMS-PCR and positive by qPCR with a value above our cutoff (>3.65%, estimated from a healthy population).

To analyze the qPCR method based on one-plus-one references against the widely used qualitative method based on ARMS-PCR [15], 20 DNA samples from patients with a suspected diagnosis of MPNs (10 positive cases and 10 determined to be negative for *JAK2* ^V617F^ using ARMS-PCR) were analyzed by qPCR in a blind experiment. The negative samples (according to ARMS-PCR) showed ABg values estimated by qPCR (mean ± standard error (SE)) of 1.9±0.6%; the ABg values of the positive samples were 55±9% (Figure 3).

Using a cutoff value of 3.65%, 18 out of 20 cases showed coincident results by both approaches. Interestingly, 2 of the 10 cases that were negative according to ARMS-PCR were positive according to qPCR, with ABg values of 5.1% and 6.7%. The most likely explanation is that these values scored below the detection limit of ARMS-PCR, which can be estimated on ABg values greater than 6.7%. Therefore, this discrepancy between the two methods may be ascribed to the greater sensitivity of qPCR. Quantitative PCR using one-plus-one template references, as a qualitative tool with a cutoff of 3.65%, allowed the identification of two false negatives by ARMS-PCR and produced no false positives.

### Two Independent Correlations Analyzes between Quantitative *JAK2* ^V617F^ ABg Determinations and the One-plus-one Reference System

**Figure 4:**
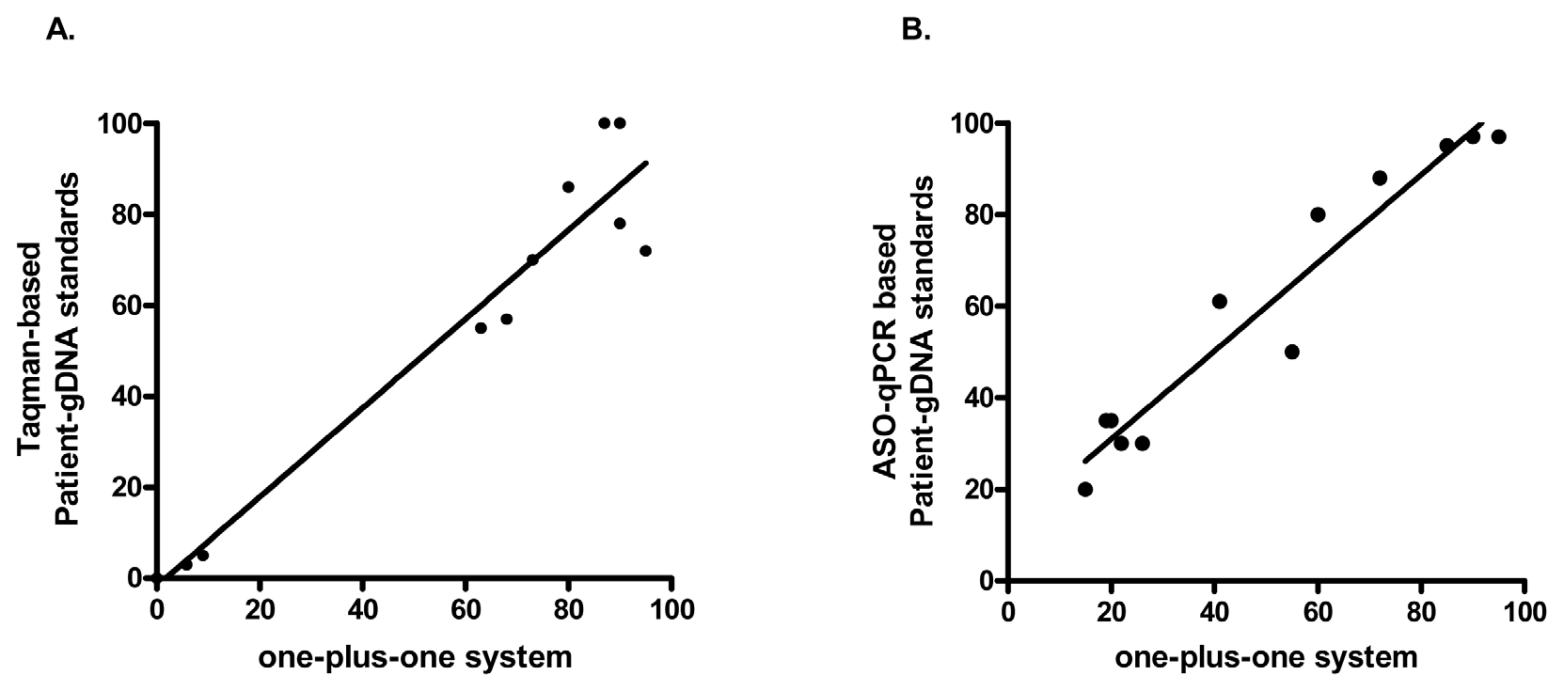
Validation of the one-plus-one reference system qPCR assay. A. Twelve MPN cases (n = 10) and negative controls (n = 2) shown highly significant (p<0.0001) correlation (r = 0.967 95%CI = 0.88–0.99) between *JAK2^V617F^* ABg measured by an allele-specific Taqman probe-based qPCR method (16) and by the one-plus-one reference system. B. Other group of 12 MPN cases shown highly significant (p<0.0001) correlation (r = 0.968 95%CI = 0.88–0.99) between *JAK2^V617F^* ABg measured by a MPN-patient/healthy-control DNA-standardized allele-specific qPCR amplification method (17) and by the one-plus-one reference system. Linear regressions are shown in both graphs.

To validate the qPCR method based on one-plus-one reference, 12 gDNA samples from patients with diagnosis of MPNs and healthy controls were subjected to a blind *JAK2^V617F^* ABg quantification using the Taqman-based method described by Bousquet et al [16]. Figure 4A shows the highly significant correlation (p<0.0001); Pearson r = 0.968 between both methods.

In addition, gDNA samples from 12 patients with MPNs were used for a second validation of the one-plus-one system against an allele-specific qPCR amplification method using a selected *JAK2^V617F^*+/+ patient and a healthy control DNA samples as standards. Equally to the other validation, Figure 4B shows a highly significant (p<0.0001) correlation (Pearson r = 0.967) between both quantitative methods.

In addition, a *JAK2*-V617F heterozygous patient-derived cell line, SET-2, was evaluated giving a *JAK2*-V617F allelic burden of 80%.

### Allele Burden and the Expression of *JAK2* ^V617F^ in Patients with MPNs

The application and performance of these new strategies of allele-specific qPCR using one-plus-one template references were tested in 19 cases with *JAK2* ^V617F^-positive MPNs: 6 PV, 5 ET and 8 PMF cases. The *JAK2* ^V617F^ allele burden and RNA expression (mean±SD) were as follows: 62.8±32.1 and 71±32.6 for PV; 53±20.6 and 53.6±21.3 for ET; and 80±14 and 97±3.4 for PMF, respectively (Table 3). This series represents preliminary results from our population and indicates a higher *JAK2* ^V617F^ allele burden and RNA expression in patients with PMF than in those with PV or ET.

**Table 3:** Allele burden and expression of *JAK2* ^V617F^ mutation.

Patient (N°)	MNP	*JAK2* ^V617F^ gAB[%]*	*JAK2* ^V617F^ cAB [%]*
1	PV	34.8	99.9
2	PV	92.6	83.4
3	PV	53.08	57.3
4	PV	19.3	12.8
5	PV	97.27	97.3
6	PV	80.3	78.6
7	ET	39.5	45.7
8	ET	67.7	45.7
9	ET	45.1	53.3
10	ET	31.5	35.02
11	ET	81.1	89.9
12	MF	86.2	99.8
13	MF	93.05	90.1
14	MF	62.3	94.1
15	MF	60.1	99.9
16	MF	98.6	99.8
17	MF	67.18	99.3
18†	MF	0.54	5.21E-04
19	MF	83.4	99.9
20	MF	91.2	95.4

* The propagated error (SD) of the AB from individual values of MT and WT measurements was negligible; therefore, it was not considered (range 6.37×10^−8^–1.5310^−5^).

† Case N° 18 was negative for the *JAK2* ^V617F^ mutation.

The patient-paired assessment of the *JAK2* ^V617F^ allele burden (ABg) and the RNA expression level (ABc) from 19 positive MPNs (Table 3) allowed us to perform a correlation analysis. A positive correlation was observed (Spearman r = 0.53, p = 0.02) even with the inclusion of four cases with increased *JAK2* ^V617F^ RNA expression levels (outliers) (Figure S5). Interestingly, all four patients in this group of outliers exhibited splenomegaly, increased white blood cell counts and bone marrow fibrosis. Although the small number of cases exhibiting *JAK2* ^V617F^ overexpression suggests that caution should be exercised concerning reaching general conclusions, this result encourages the performance of further studies.

## Discussion

The discovery of mutations in *JAK2* has allowed crucial advances in the understanding of the pathophysiology of typical *BCR-ABL1*-negative MPNs. The mutation *JAK2* ^V617F^ is a useful molecular marker that has improved and simplified the diagnosis of these disorders. The *JAK2* ^V617F^ mutation is found in more than 90% of patients with PV and in nearly one-half of those with PMF or ET [17], [18]. Consequently, all the recommended diagnostic algorithms for these entities include qualitative molecular information regarding *JAK2* mutations [19]. However, a quantitative study stratifying patients into different quartiles according to their allele burden at diagnosis may be even more appropriate for evaluating the clinical implications of *JAK2* ^V617F^ load.

A multicenter study demonstrated large discrepancies between the different methods used to quantify the *JAK2* ^V617F^ mutation [20]. Hence, it is extremely important to employ suitable reference standards to allow an exact quantification of the *JAK2* ^V617F^ allele burden.

Considering that a blood leukocyte sample represents a potential mixture of cells that are homo/heterozygous for *JAK2* ^V617F^, homozygosity cannot be determined when the allele burden is lower than 50% and it can only be warranted when the proportion of the *JAK2* ^V617F^ allele is significantly greater than 50%. Because the presence of a *JAK2* ^V617F^ homozygous clone is associated with major clinical consequences, it is crucial to determine the AB turning point (i.e., 50%) without bias.

In addition, a method that permits the exact and reproducible quantification of *JAK2* ^V617F^ is extremely valuable for the evaluation of patients with MPNs, particularly for the follow-up of patients treated with JAK2 inhibitors.

There is a growing interest in assessing the *JAK2* ^V617F^ allele burden and in its potential influence on disease phenotype, disease complications and evolution [11]; raising the possibility that homozygosity for the mutant allele is a time-dependent clonal evolution event [4]. The use of different reference standards for quantitative assays may generate discrepancies between AB values.

We provided two independent validations comparing the one-plus-one plasmid-based method with an allele-specific Taqman-probe based qPCR method [16]; and with a method based on curves made from patient samples, using a V617F *JAK2* homozygous patient and a *JAK2* non-mutated control, as has been used in a number of laboratories worldwide [20]. Recently, the European Leukemia Net (ELN) performed a study for establishing optimal quantitative-polymerase chain reaction assays for routine diagnosis of *JAK2*-V617F by comparing 12 laboratories: three of them using unpublished ‘in-house’ developed assays and nine of them applying published standard curves using either independently measured plasmid DNA for *JAK2*-WT and *JAK2*-V617F or, alternatively, DNA samples from a homozygous *JAK2*-V617 patient and a healthy donor [21]. Quentmeier et al revealed an active mitotic recombination on *JAK2*-V617F positive cell lines such as MB-02, MUTZ8, HEL or SET-2 using FISH (fluorescent *in situ* hybridization) and microsatellite analysis, which associates with genetic imbalances on *JAK2 locus* and may cause quantification inconsistencies when these cell lines are used for standard curves [22]. In agree with this evidence, we measured an allelic burden of 80% from SET-2, a *JAK2^V617F^* heterozygous patient-derived cell line, reflecting an active mitotic recombination *in vitro* and the lack of reliability to use it for standard curves.

The quantification method presented in this paper would be most appropriate for assessing ABs of approximately 50% because the molecular structure of the construct (one-plus-one) warrants a fixed 1∶1 ratio between the mutated and wild-type *JAK2* PCR templates. To the best of our knowledge, no standard for real-time PCR-based quantitative approaches has used the one-plus-one template structure thus far.

As a qualitative tool, our approach using a threshold value (obtained in healthy donors) of 3.65% (mean +2×SD) allowed the positive molecular detection of *JAK2* ^V617F^ in 19 cases with MPNs and demonstrated a more sensitive detection limit than ARMS-PCR (≥6.7%).

This qPCR-based approach using one-plus-one template references allowed the rapid estimation of the allele burden and RNA expression of *JAK2* ^V617F^ in 19 positive cases with classical MPNs and detected 13 cases associated with homozygous clones (ABg≥56.73% [mean+SD]).

Although the sample size prevents general conclusions about Argentinian patients with MPNs, a similar trend to those reported in the literature for the *JAK2* ^V617F^ allele was observed in our group: higher ABg and ABc expression in patients with PMF or PV than in patients with ET.

Although the relative expression level of *JAK2* ^V617F^ was variable, this depends mainly on the percentage of ABg in the majority of cases. We observed correlations between the levels of *JAK2* ^V617F^ ABg and ABc in patients with PV, ET and PMF, in agreement with the results reported by Lippert *et al.* [23] and Tiedt *et al.* [24]. In contrast to the general trend, we found four outliers (i.e., patients with relatively increased levels of *JAK2* ^V617F^ transcripts) (Figure S5) who exhibited splenomegaly, high white blood counts and bone marrow fibrosis. The possibility of *JAK2* ^V617F^ allele overexpression or differential RNA stability in MPNs and the possible clinical consequences are extremely interesting points that merit further investigation.

In conclusion, the qPCR method using one-plus-one template references reported here for *JAK2* ^V617F^ allele quantification represents a cost-effective tool that is particularly appropriate for measuring the critical AB associated with the transition to the homozygosity state, which is of prognostic value in classical MPN cases.

## Supporting Information

Figure S1 ***JAK2*** **V617F MT::WT 1::1 reference PCR construction strategy. A.** gDNA. The first series of PCR amplifications was performed to obtain products (i), (ii) and (iii) from genomic DNA substrates (references in the gDNA-construct section of the main text). The second series produced (iv) and (v) from PCR substrates (i) plus (ii) and (ii) plus (iii), respectively. The third series produced the full-length gDNA *JAK2* V617F MT::WT 1::1 reference construct. The primers and DNA substrates for PCR amplification are indicated. **B.** cDNA. The first series of PCR amplifications was performed to obtain products (i’), (ii’) and (iii’) from complementary DNA (randomly primed, reverse-transcribed total RNA) substrates (references in the cDNA construct section of the main text). The second series produced (iv’) from substrates (i’) plus (ii’) and (v’) from substrates (ii’) plus (iii’). The third series produced the full-length cDNA *JAK2* V617F MT::WT 1::1 reference construct by fusing PCR products (iv’) and (v’). The primers and DNA substrates for each PCR amplification are indicated.
(PPT)

Figure S2 **cDNA and gDNA** ***JAK2*** **V617F MT::WT 1::1 PCR construction steps and MT/WT analysis by DNA sequencing and BsaXI restriction enzyme digestion. A.** Agarose gel electrophoresis of all the PCR products and the final product, the *JAK2* MT/WT 1::1 gDNA construct. (1) amplimer of *JAK2* gDNA V617F MT-arm (453 bp), (2) amplimer of *JAK2* gDNA V617 WT-arm (453 bp), (3) DNA-spacer (473 bp, *F8* gene part of IVS22), (4) fusion amplimer of (1) (MT-arm), plus (3) (spacer) (775 bp), (5) fusion amplimer of (2) (WT-arm), plus (3) (spacer) (781 bp), (6) final fusion amplimer of (4) (MT-arm+spacer) plus (5) (WT-arm+spacer) (1083 bp). **B.** Agarose gel electrophoresis of all the PCR products and the final product, the *JAK2* MT/WT 1::1 cDNA construct. M indicates 100-bp ladder molecular marker. (1) amplimer of *JAK2* cDNA V617F MT-arm (371 bp), (2) amplimer of *JAK2* cDNA V617 WT-arm (371 bp), (3) DNA-spacer (473 bp, *F8* gene part of IVS22), (4) fusion amplimer of (1) (MT-arm) plus (3) (spacer) (752 bp) and (5) final fusion amplimer of (4) (MT-arm+spacer) plus (spacer+WT-arm) (1023 bp). The final cDNA and gDNA constructs (i.e., A. [6] and B. [5]) were cloned and DNA sequenced. **C** and **D** show the relevant DNA sequences of the WT-arm (upper panel) and MT-arm (lower panel) of the gDNA and cDNA recombinant plasmids, respectively. **E.** Agarose gel electrophoresis showing the BsaXI restriction analysis of both constructs: (1) undigested gDNA, (2) BsaXI-digested gDNA, (3) undigested cDNA and (4) BsaXI-digested cDNA.
(PPT)

Figure S3 **Experiments to check the structural specificity of the gDNA (A) and cDNA (B) reference plasmids.** The experimental results are shown by the agarose gel electrophoresis analysis of the PCR products. The annealing temperatures used in the PCR amplification and the combined primer pairs are indicated at the top and bottom of each gel image, respectively.
(PPT)

Figure S4 ***JAK2*** **^V617F^ and** ***JAK2*** **^WT^ DNA standard curves. A.** cDNA. The upper curves show the PCR amplification cycle versus the fluorescence (530 nm) from triplicates of serial dilutions (i.e., 10^−5^, 10^−6^, 10^−7^ and 10^−8^) of the *JAK2* cDNA MT:WT 1∶1 plasmid. The lower graphs show the corresponding log-transformed standard curves of the cDNA-plasmid concentration (arbitrary units, **AUc** associated with a specific dilution of the same plasmid) versus the crossing points for the *JAK2* ^V617F^ mutation (left) and *JAK2* ^WT^ (right), as indicated. Eff. indicates the efficiency of the real-time PCR amplification. Note that standard curves share the same cDNA-plasmid concentration units (**AUc**); therefore, these units may be added or canceled in relative quantification equations. **B.** gDNA. The upper curves show the PCR amplification cycle versus the fluorescence (530 nm) from triplicates of serial dilutions (i.e., 10^−3^, 10^−4^, 10^−5^, 10^−6^ and 10^−7^) of the *JAK2* gDNA MT:WT 1∶1 plasmid. The lower graphs show the corresponding log-transformed standard curves of the gDNA-plasmid concentration (arbitrary units, **AUg** associated with a specific dilution of the same plasmid) versus the crossing point for the *JAK2* ^V617F^ mutation (left) and *JAK2^WT^* (right), as indicated. Eff. indicates the efficiency of the real-time PCR amplification. Again, the standard curves share the same plasmid concentration units (**AUg**); therefore, these may be added or canceled in relative quantification equations.
(PPT)

Figure S5 **Correlation analysis between qPCR results using gDNA and cDNA as substrates.** There was a significant correlation between the allelic burden and expression levels of the JAK2^V617F^ mutation (Spearman P<0.0002). Four cases with increased *JAK2* ^V617F^ RNA expression levels (outliers) are indicated.
(PPT)

Methods S1 (DOC)
