# Supplementary material for: Improved Diagnosis of the Transition to JAK2 V617F Homozygosity: The Key Feature for Predicting the Evolution of Myeloproliferative Neoplasms
Source: PLoS One. 2014 Jan 27;9(1):e86401. doi: 10.1371/journal.pone.0086401 (PMC3903535; doi:10.1371/journal.pone.0086401)
Supplement: Figure S1 — JAK2 V617F MT::WT 1::1 reference PCR construction strategy. A. gDNA. The first series of PCR amplifications was performed to obtain products (i), (ii) and (iii) from genomic DNA substrates (references in the gDNA-construct section of the main text). The second series produced (iv) and (v) from PCR substrates (i) plus (ii) and (ii) plus (iii), respectively. The third series produced the full-length gDNA JAK2 V617F MT::WT 1::1 reference construct. The primers and DNA substrates for PCR amplification are indicated. B. cDNA. The first series of PCR amplifications was performed to obtain products (i’), (ii’) and (iii’) from complementary DNA (randomly primed, reverse-transcribed total RNA) substrates (references in the cDNA construct section of the main text). The second series produced (iv’) from substrates (i’) plus (ii’) and (v’) from substrates (ii’) plus (iii’). The third series produced the full-length cDNA JAK2 V617F MT::WT 1::1 reference construct by fusing PCR products (iv’) and (v’). The primers and DNA substrates for each PCR amplification are indicated. (PPT) [file pone.0086401.s001.ppt]

## Slide 1
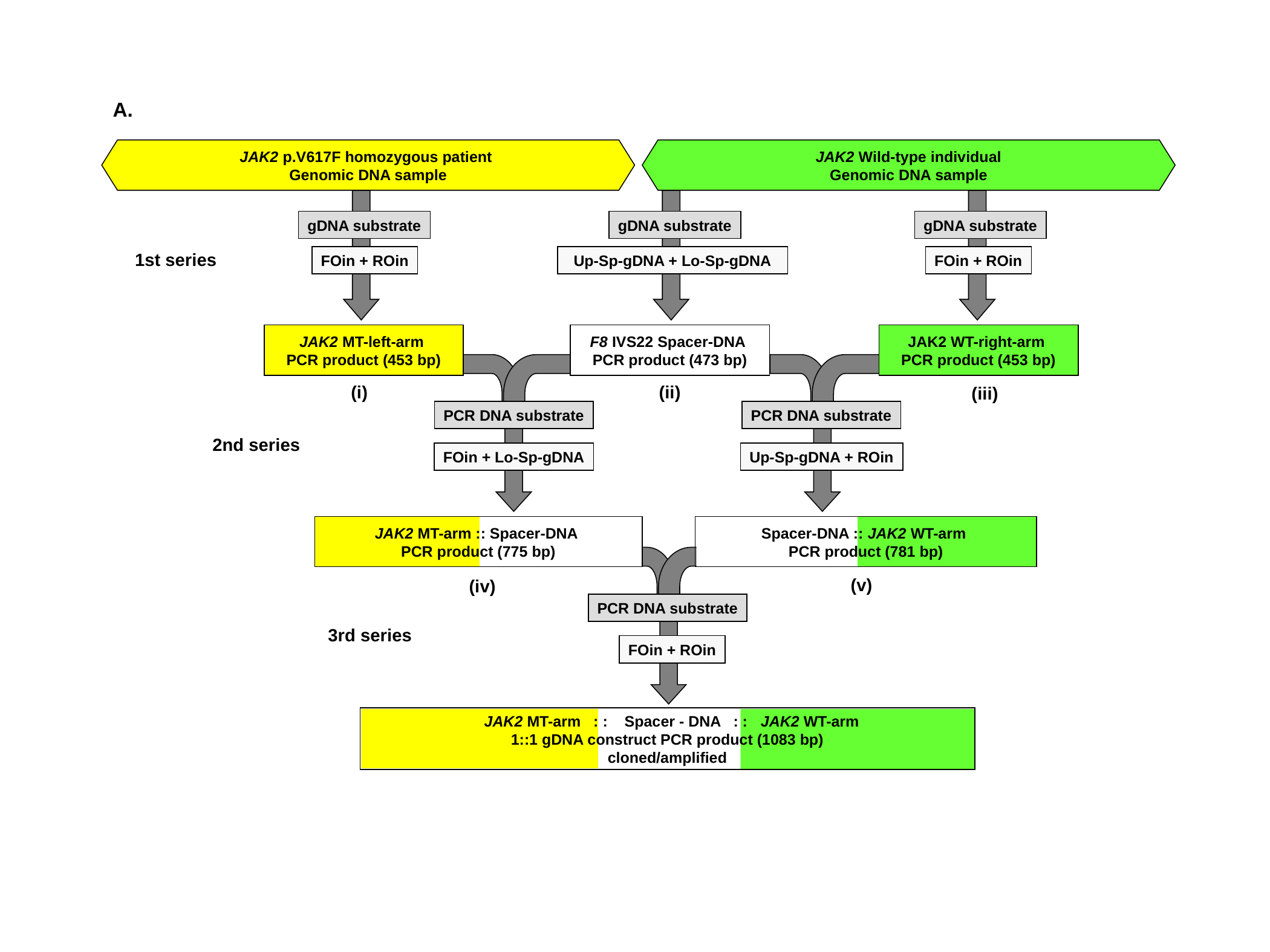

A.
JAK2 p.V617F homozygous patient
Genomic DNA sample
JAK2 Wild-type individual
Genomic DNA sample
gDNA substrate
gDNA substrate
gDNA substrate
FOin + ROin
Up-Sp-gDNA + Lo-Sp-gDNA
FOin + ROin
JAK2 MT-left-arm
PCR product (453 bp)
F8 IVS22 Spacer-DNA
PCR product (473 bp)
JAK2 WT-right-arm
PCR product (453 bp)
PCR DNA substrate
PCR DNA substrate
FOin + Lo-Sp-gDNA
Up-Sp-gDNA + ROin
JAK2 MT-arm :: Spacer-DNA
PCR product (775 bp)
Spacer-DNA :: JAK2 WT-arm
PCR product (781 bp)
PCR DNA substrate
FOin + ROin
 JAK2 MT-arm : : Spacer - DNA : : JAK2 WT-arm
1::1 gDNA construct PCR product (1083 bp)
cloned/amplified
1st series
(ii)
(i)
(iii)
2nd series
(v)
(iv)
3rd series

## Slide 2
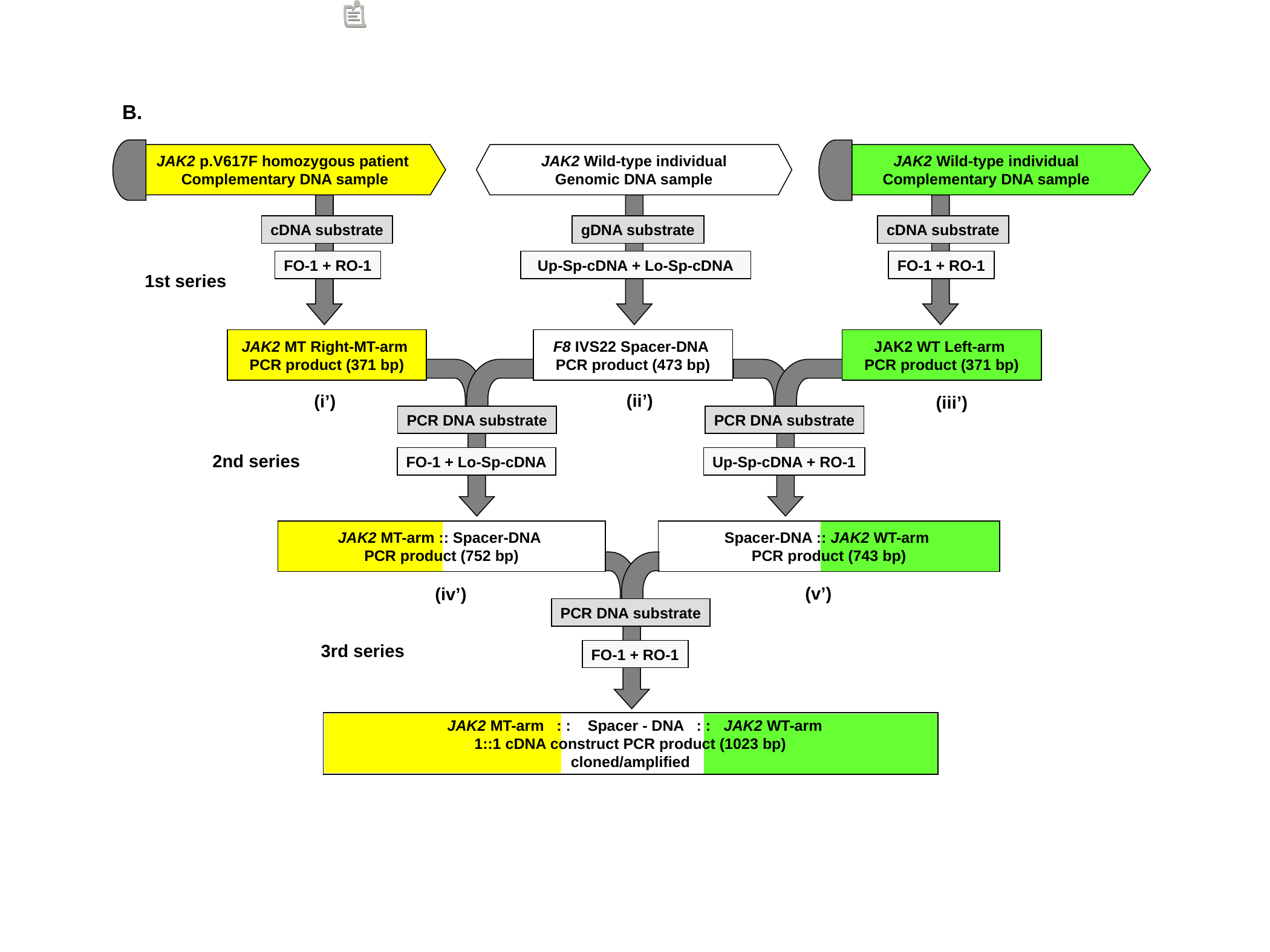

B.
JAK2 p.V617F homozygous patient
Complementary DNA sample
JAK2 Wild-type individual
Genomic DNA sample
JAK2 Wild-type individual
Complementary DNA sample
cDNA substrate
gDNA substrate
cDNA substrate
FO-1 + RO-1
Up-Sp-cDNA + Lo-Sp-cDNA
FO-1 + RO-1
JAK2 MT Right-MT-arm
PCR product (371 bp)
F8 IVS22 Spacer-DNA
PCR product (473 bp)
JAK2 WT Left-arm
PCR product (371 bp)
PCR DNA substrate
PCR DNA substrate
FO-1 + Lo-Sp-cDNA
Up-Sp-cDNA + RO-1
JAK2 MT-arm :: Spacer-DNA
PCR product (752 bp)
Spacer-DNA :: JAK2 WT-arm
PCR product (743 bp)
PCR DNA substrate
FO-1 + RO-1
 JAK2 MT-arm : : Spacer - DNA : : JAK2 WT-arm
1::1 cDNA construct PCR product (1023 bp)
cloned/amplified
1st series
(ii’)
(i’)
(iii’)
2nd series
(v’)
(iv’)
3rd series
