# Supplementary figures and images for: Improved Diagnosis of the Transition to JAK2 V617F Homozygosity: The Key Feature for Predicting the Evolution of Myeloproliferative Neoplasms
Source: PLoS One. 2014 Jan 27;9(1):e86401. doi: 10.1371/journal.pone.0086401 (PMC3903535; doi:10.1371/journal.pone.0086401)

## Slide 1
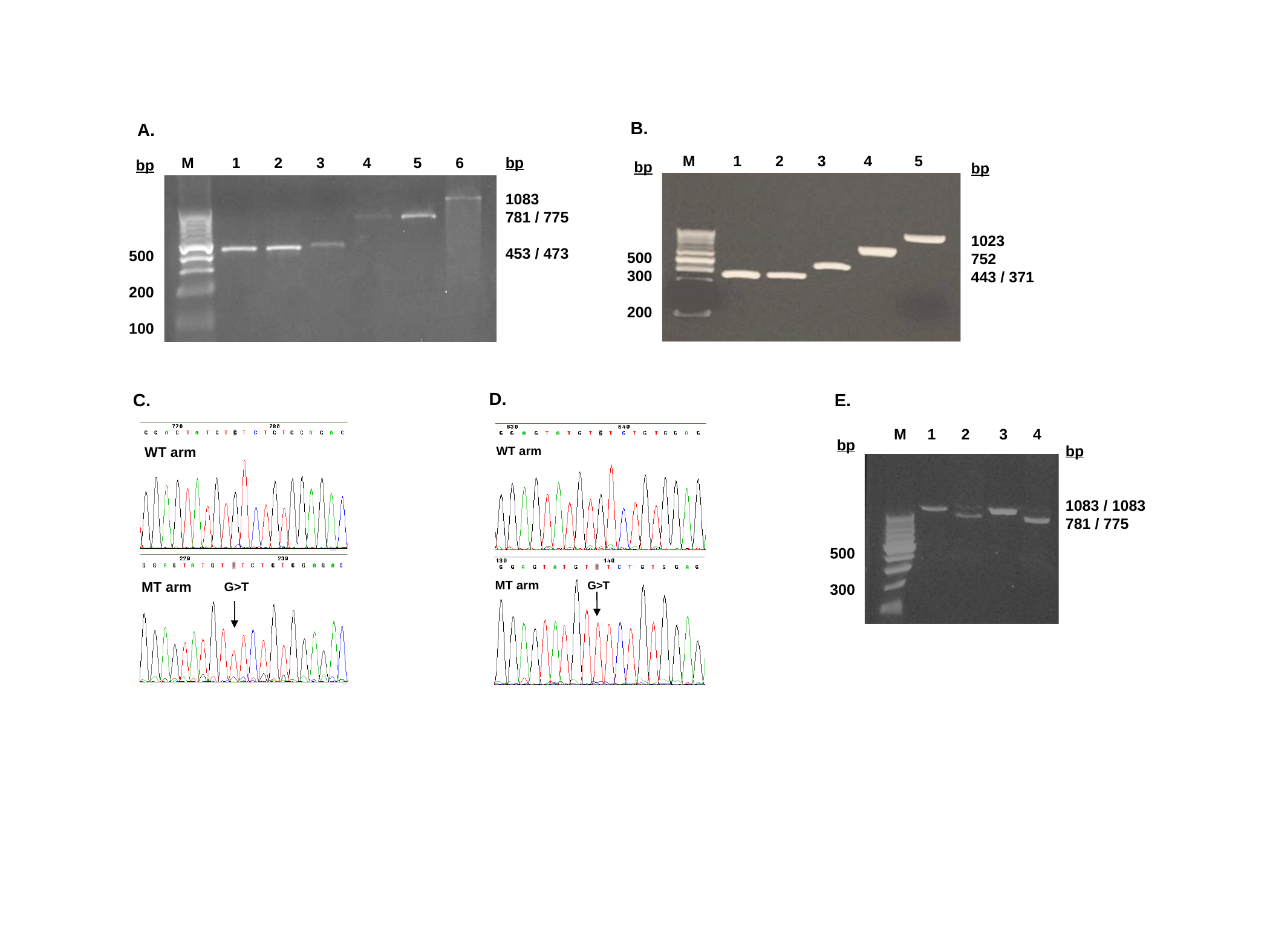

B.
A.
M 1 2 3 4 5
bp
500
300
200
bp
1023
752
443 / 371
bp
1083
781 / 775
453 / 473
M 1 2 3 4 5 6
bp
500
200
100
D.
C.
E.
M 1 2 3 4
bp
500
300
bp
1083 / 1083
781 / 775
WT arm
MT arm
G>T
WT arm
MT arm
G>T

Supplement: Figure S2 — cDNA and gDNA JAK2 V617F MT::WT 1::1 PCR construction steps and MT/WT analysis by DNA sequencing and BsaXI restriction enzyme digestion. A. Agarose gel electrophoresis of all the PCR products and the final product, the JAK2 MT/WT 1::1 gDNA construct. (1) amplimer of JAK2 gDNA V617F MT-arm (453 bp), (2) amplimer of JAK2 gDNA V617 WT-arm (453 bp), (3) DNA-spacer (473 bp, F8 gene part of IVS22), (4) fusion amplimer of (1) (MT-arm), plus (3) (spacer) (775 bp), (5) fusion amplimer of (2) (WT-arm), plus (3) (spacer) (781 bp), (6) final fusion amplimer of (4) (MT-arm+spacer) plus (5) (WT-arm+spacer) (1083 bp). B. Agarose gel electrophoresis of all the PCR products and the final product, the JAK2 MT/WT 1::1 cDNA construct. M indicates 100-bp ladder molecular marker. (1) amplimer of JAK2 cDNA V617F MT-arm (371 bp), (2) amplimer of JAK2 cDNA V617 WT-arm (371 bp), (3) DNA-spacer (473 bp, F8 gene part of IVS22), (4) fusion amplimer of (1) (MT-arm) plus (3) (spacer) (752 bp) and (5) final fusion amplimer of (4) (MT-arm+spacer) plus (spacer+WT-arm) (1023 bp). The final cDNA and gDNA constructs (i.e., A. [6] and B. [5]) were cloned and DNA sequenced. C and D show the relevant DNA sequences of the WT-arm (upper panel) and MT-arm (lower panel) of the gDNA and cDNA recombinant plasmids, respectively. E. Agarose gel electrophoresis showing the BsaXI restriction analysis of both constructs: (1) undigested gDNA, (2) BsaXI-digested gDNA, (3) undigested cDNA and (4) BsaXI-digested cDNA. (PPT) [file pone.0086401.s002.ppt]

## Slide 1
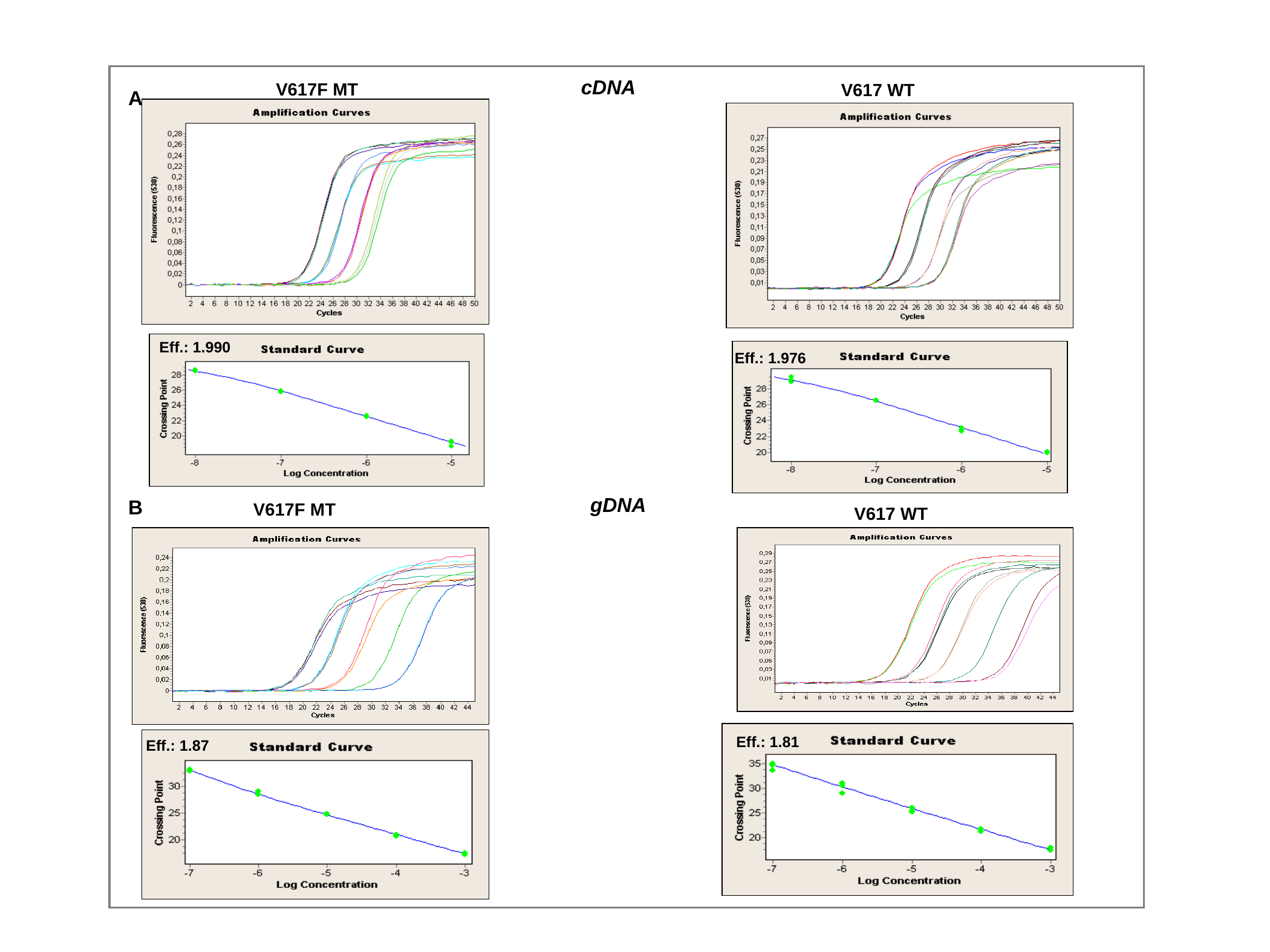

cDNA
V617F MT
V617 WT
A
Eff.: 1.990
Eff.: 1.976
gDNA
B
V617F MT
V617 WT
Eff.: 1.81
Eff.: 1.87

Supplement: Figure S4 — JAK2 V617F and JAK2 WT DNA standard curves. A. cDNA. The upper curves show the PCR amplification cycle versus the fluorescence (530 nm) from triplicates of serial dilutions (i.e., 10−5, 10−6, 10−7 and 10−8) of the JAK2 cDNA MT:WT 1∶1 plasmid. The lower graphs show the corresponding log-transformed standard curves of the cDNA-plasmid concentration (arbitrary units, AUc associated with a specific dilution of the same plasmid) versus the crossing points for the JAK2 V617F mutation (left) and JAK2 WT (right), as indicated. Eff. indicates the efficiency of the real-time PCR amplification. Note that standard curves share the same cDNA-plasmid concentration units (AUc); therefore, these units may be added or canceled in relative quantification equations. B. gDNA. The upper curves show the PCR amplification cycle versus the fluorescence (530 nm) from triplicates of serial dilutions (i.e., 10−3, 10−4, 10−5, 10−6 and 10−7) of the JAK2 gDNA MT:WT 1∶1 plasmid. The lower graphs show the corresponding log-transformed standard curves of the gDNA-plasmid concentration (arbitrary units, AUg associated with a specific dilution of the same plasmid) versus the crossing point for the JAK2 V617F mutation (left) and JAK2WT (right), as indicated. Eff. indicates the efficiency of the real-time PCR amplification. Again, the standard curves share the same plasmid concentration units (AUg); therefore, these may be added or canceled in relative quantification equations. (PPT) [file pone.0086401.s004.ppt]

## Slide 1
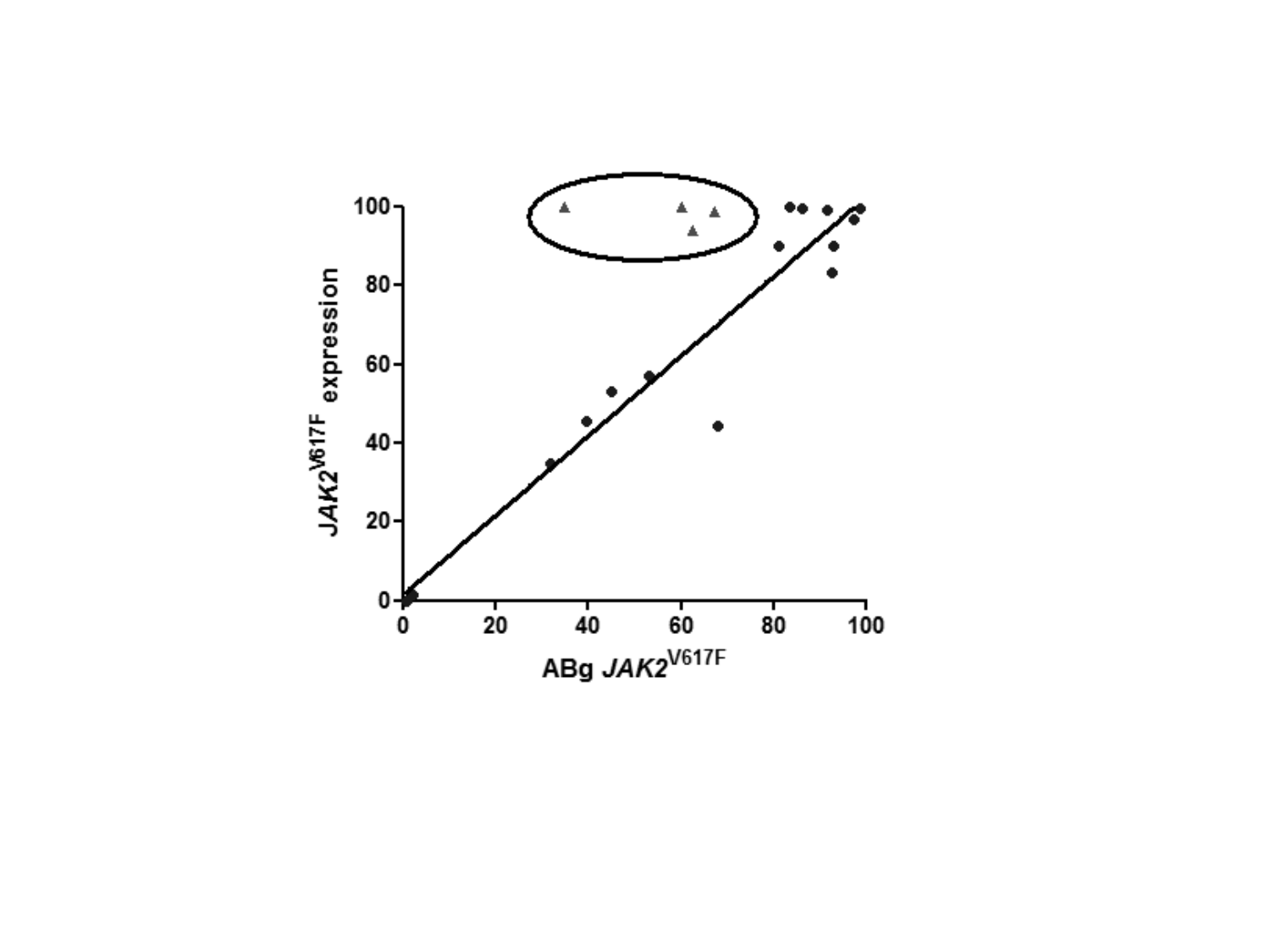

Supplement: Figure S5 — Correlation analysis between qPCR results using gDNA and cDNA as substrates. There was a significant correlation between the allelic burden and expression levels of the JAK2V617F mutation (Spearman P<0.0002). Four cases with increased JAK2 V617F RNA expression levels (outliers) are indicated. (PPT) [file pone.0086401.s005.ppt]
