# Supplementary material for: Improved Diagnosis of the Transition to JAK2 V617F Homozygosity: The Key Feature for Predicting the Evolution of Myeloproliferative Neoplasms
Source: PLoS One. 2014 Jan 27;9(1):e86401. doi: 10.1371/journal.pone.0086401 (PMC3903535; doi:10.1371/journal.pone.0086401)
Supplement: Figure S3 — Experiments to check the structural specificity of the gDNA (A) and cDNA (B) reference plasmids. The experimental results are shown by the agarose gel electrophoresis analysis of the PCR products. The annealing temperatures used in the PCR amplification and the combined primer pairs are indicated at the top and bottom of each gel image, respectively. (PPT) [file pone.0086401.s003.ppt]

## Slide 1
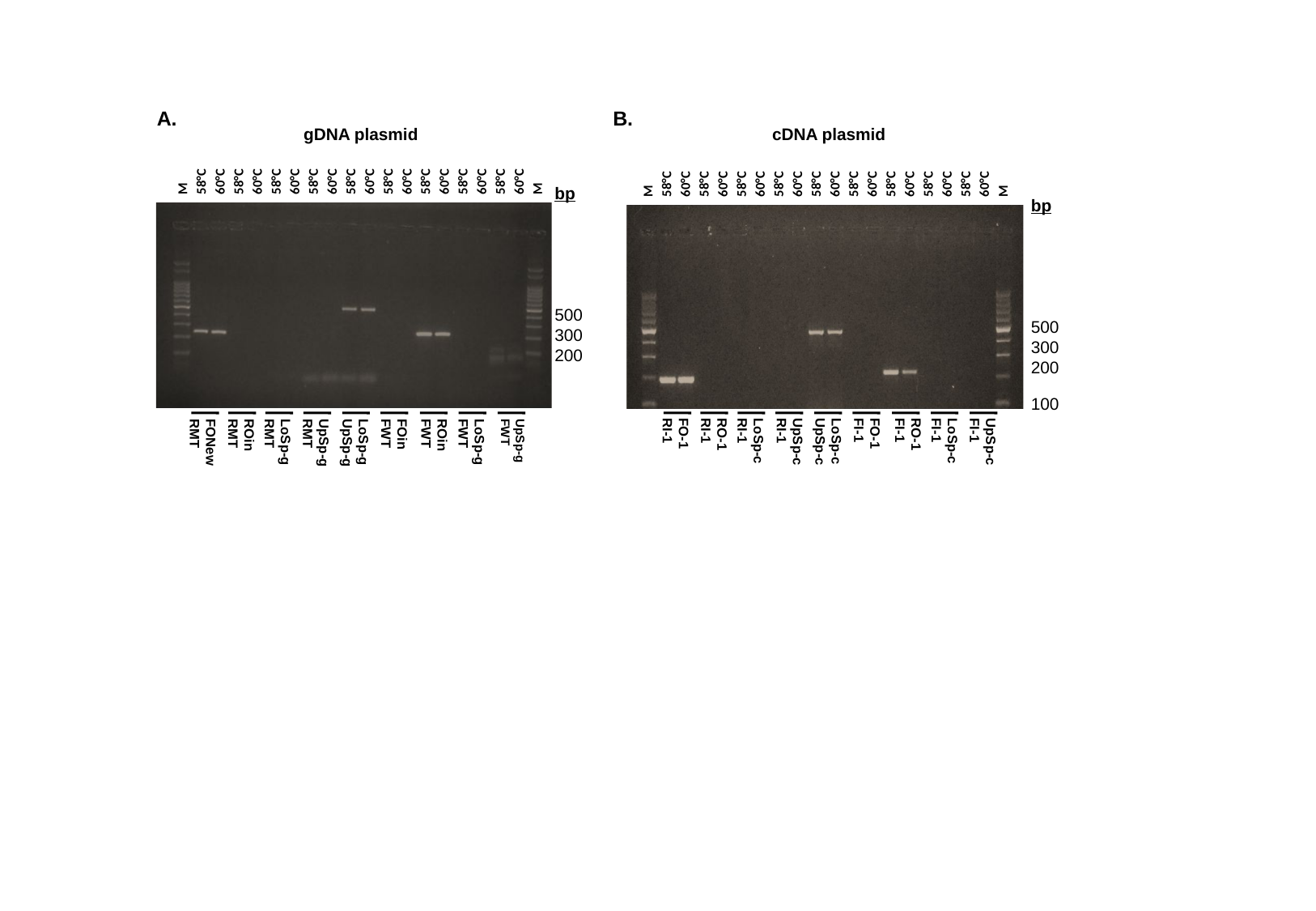

M
58°C
60°C
58°C
60°C
58°C
60°C
58°C
60°C
58°C
60°C
58°C
60°C
58°C
60°C
58°C
60°C
58°C
60°C
M
M
58°C
60°C
58°C
60°C
58°C
60°C
58°C
60°C
58°C
60°C
58°C
60°C
58°C
60°C
58°C
60°C
58°C
60°C
M
A.
B.
gDNA plasmid
cDNA plasmid
bp
500
300
200
bp
500
300
200
100
FO-1
RI-1
RO-1
RI-1
FO-1
FI-1
RO-1
FI-1
ROin
RMT
FOin
FWT
ROin
FWT
FONew
RMT
LoSp-c
RI-1
UpSp-c
RI-1
LoSp-c
UpSp-c
LoSp-c
FI-1
UpSp-c
FI-1
LoSp-g
RMT
UpSp-g
RMT
LoSp-g
UpSp-g
LoSp-g
FWT
UpSp-g
FWT
