# Supplementary material for: Improved Diagnosis of the Transition to JAK2 V617F Homozygosity: The Key Feature for Predicting the Evolution of Myeloproliferative Neoplasms
Source: PLoS One. 2014 Jan 27;9(1):e86401. doi: 10.1371/journal.pone.0086401 (PMC3903535; doi:10.1371/journal.pone.0086401)
Supplement: Methods S1 — (DOC) [file pone.0086401.s006.doc]

**Supporting Methods S1**

The strategies used to assemble the gDNA and cDNA *JAK2*V^617F^-*JAK2*^WT^ one-plus-one constructs are shown in Supporting Figure S1 A and B, respectively. The intermediate and final PCR products for the gDNA and cDNA reference constructs are presented in Supporting Figure S2 A and B, respectively. The oligonucleotide primers used to amplify these constructs are listed in Table 2.

*Assembly of* JAK2 *gDNA MT::WT 1::1 reference construct:*

Three PCR products were created by a first series of PCR amplifications: (i) an MT-left-arm (453 bp) was amplified from a gDNA sample from a *JAK2*^V617F^ virtually homozygous patient using the FOin and ROin primers (table 2); (ii) a Spacer-DNA (473 bp) was amplified by targeting a region of *F8* intron 22 using the Up-Sp-gDNA and Lo-Sp-gDNA primers, each designed with 15-nucleotide 5’ tails to allow fusions with the arms (table 2); and (iii) a WT-right-arm (453 bp) was amplified from a gDNA sample from an individual with a *JAK2*^WT^ genotype using the FOin and ROin primers (Supporting Figure S1 A).

A second series of PCR amplifications served to perform two fusions: the MT-left-arm obtained in (i) was fused with the Spacer-DNA from (ii) using the FOin and Lo-Sp-gDNA primers to create a 775-bp product (iv), and the WT-right-arm obtained in (iii) was fused with the Spacer-DNA from (ii) using the Up-Sp-gDNA and ROin primers to create a 781-bp product (v).

All the primer tails for fusion (Up-Sp-gDNA and Lo- Sp-gDNA) and the fusion primers used to construct the cDNA reference (Up-Sp-cDNA and Lo- Sp-cDNA; see the following section) were designed to target restricted sequences. For example, a 5’-excluded vicinal T (thymidine) was employed to enable the proper annealing of PCR product strands with 3’ overhanging As (adenines) that had been added by Taq polymerase.

A third series of PCR amplifications was performed to fuse the products obtained in (iv) and (v), which were used as a substrate with the FOin and ROin primers, to produce the final version of the *JAK2* gDNA-MT::WT reference (1083 bp) (Figure 1 A, Supporting Figure S1 A). Note that the PCR products from (iv) and (v) overlapped 473 nucleotides (including the tails) of the 3’ end complementary strands to enable the fusion.

To assemble the *JAK2* gDNA-MT::WT 1::1 reference, PCR was performed to amplify a leukocyte-extracted gDNA substrate under conditions similar to those used in the first series of three separate amplifications (which produced the (i) MT-left-arm, (ii) Spacer-DNA and (iii) WT-right-arm). Briefly, the reactions were performed in a total volume of 25 μl with 150 ng of gDNA substrate, 0.4 μM of each primers, 200 μM of dNTPs (Invitrogen, Argentina), 1 unit of GoTaq DNA Polymerase, the corresponding GoTaq buffer and 1.5 mM MgCl_2_ (Promega, Argentina). The thermocycling conditions (touchdown PCR) for the MT-left-arm, Spacer-DNA and WT-right-arm consisted of 94° C for 2 min; four cycles of 94° C for 45 sec, 60° C for 1 min and 72° C for 90 sec; 24 cycles of 94° C for 45 sec, 55° C for 1 min and 72° C for 90 sec; and a final extension step at 72° C for 5 min.

A second series of PCR amplifications produced two fusions: (iv) MT-left-arm with Spacer-DNA and (v) Spacer-DNA with WT-right-arm. To perform these fusions, 2 μl of diluted and column-purified PCR products were amplified with reagents similar to those described above. The following touchup PCR conditions were employed due to the reduced homology between the products (15 nucleotides introduced by Spacer primer tails, table 2): 94°C for 2 min; 4 cycles of 94°C for 45 sec, 50°C for 1 min and 72°C for 2 min; 24 cycles of 94°C for 45 sec, 55°C for 1 min and 72°C for 2 min; and a final extension step at 72°C for 5 min.

The final, 1083-bp gDNA MT::WT construct was amplified by fusing 2 μl of diluted and column-purified PCR products (iv) and (v) using the FOin and ROin primers (Table 2) under conditions similar to those described above but with 3 units of GoTaq DNA Polymerase. A modified touchdown PCR protocol was applied: 95°C for 3 min; 10 cycles of 95°C for 1 min and 72°C for 3 min; 10 cycles of 94°C for 45 sec, 60°C for 1 min and 72°C for 5 min; 10 cycles of 94°C for 45 sec, 55°C for 1 min and 72°C for 7 min; and a final extension step at 72°C for 5 min.

The successful amplification of the PCR products (i, ii, iii, iv, v and the final gDNA reference) was confirmed by ethidium bromide-stained agarose gel (1.5-2%) electrophoresis (Supporting Figure S2 A). The PCR products were excised from the agarose gel and cleaned with the QIAquick PCR purification kit (Qiagen, Valencia, CA) for subsequent use.

*Assembly of* JAK2 *cDNA MT::WT 1::1 reference construct:*

To assemble the cDNA MT::WT reference, three PCR products were created by a first series of PCR amplifications: (i’) an MT-left-arm (371 bp) was amplified from 3 μl of a cDNA sample from a *JAK2*^V617F^ homozygous patient using the FO-1 and RO-1 primers (Table 2); (ii’) a Spacer-DNA (473 bp) was amplified by targeting a region of *F8* intron 22 with the Up-Sp-cDNA and Lo-Sp-cDNA primers, each designed with a 15-nucleotide 5’ tail to enable fusions with the MT-left-arm and WT-right-arm, respectively (Table 2); and (iii’) a WT-right-arm (371 bp) was amplified from 3 μl of a cDNA sample from an individual with a *JAK2*^WT^ genotype using the FO-1 and RO-1 primers (Supporting Figure S1 B). The reagent and primer concentrations and the thermocycling conditions used to amplify (i’), (ii’) and (iii’) were identical to those applied to amplify the gDNA construct, i.e., (i), (ii) and (iii).

For the construction of the cDNA MT::WT reference, a second series of PCR amplifications produced the fusion of (i’) MT-left-arm with (ii’) Spacer-DNA to form a 752-bp product (iv’) and the fusion of (ii’) Spacer-DNA with (iii’) WT-right-arm to form a 743-bp product (v’). These two fusions were performed using 2 μl of diluted and column-purified PCR products with reagents similar to those described above and the same touchup PCR thermocycling conditions.

The final, 1023-bp cDNA MT::WT reference was amplified by fusing 2 μl of diluted and column-purified PCR products (iv’) and (v’) (obtained in the second series of PCR amplifications) using the FO-1 and RO-1 primers (Table 2). The PCR reagents, conditions and thermocycling conditions were the same as those described above for the final gDNA reference.

The intermediate PCR products for the cDNA construct, (i’), (ii’), (iii’), (iv’) and the final cDNA MT::WT reference were analyzed by agarose gel electrophoresis (Supporting Figure S2 B), excised from the agarose gel and prepared for subsequent manipulations using the QIAquick PCR purification kit (Qiagen).

*Plasmid cloning of the* JAK2 *gDNA and cDNA MT::WT 1::1 reference standards for amplification and storage*

Aliquots (4 μl) of the amplified PCR products of the two constructs (i.e., cDNA and gDNA) were directly ligated into the pCR2.1-TOPO plasmid vector at room temperature for 5 min under the conditions recommended by the plasmid provider (Invitrogen SRL, Argentina).

To improve the transformation efficiency and final plasmid copy number, 1 μl of the TOPO cloning reaction was added to transform One Shot DH5αTM-T1 competent *E. coli* cells under the conditions recommended by the manufacturer (Invitrogen).

Competent *E. coli* cells for the transformation reactions were incubated on ice for 15 min and then heat-shocked for 30 sec at 42°C. The tubes were immediately chilled on ice with 250 μl of room-temperature S.O.C. medium, capped and shaken horizontally at 250 rpm at 37°C for 1 hour. Aliquots (20 μl) of the transformed cells were grown overnight at 37°C on selective plates with 50 mg/ml ampicillin and 40 μl of X-gal. The chromogenic substrate X-gal was used to enable the separation of colonies with the insert from colonies without an insert by blue-white screening. The plates with colonies were stored at 4°C. Ten white colonies were grown in 2 ml of LB medium with 50 mg/ml ampicillin at 37°C overnight. The cultures were then centrifuged, and the bacterial cells were resuspended in 250 μl of alkaline lysis buffer to preferentially release plasmid DNA over chromosomal DNA. After centrifugation for 10 minutes at 13,000 rpm, the plasmid DNA-enriched supernatants were transferred to QIAprep spin columns (Qiagen), washed twice and eluted with 50 μl of elution buffer into 1.5-ml tubes. The eluted DNA was stored at 4°C until further use.

The DNA copy number of the plasmid preparations (molecules/µl) was estimated by dividing the absorbance at 260 nm (g/µl) by ([bp plasmid + bp construct] x 660 daltons / Avogadro’s number) (g/molecule).
